# The timing of childhood adversity associates with epigenetic patterns across childhood and adolescence: results from a prospective, longitudinal study

**DOI:** 10.1016/S2352-4642(23)00127-X

**Published:** 2023-06-14

**Authors:** Alexandre A. Lussier, Yiwen Zhu, Brooke J. Smith, Janine Cerutti, Jonah Fisher, Phillip Melton, Natasha M. Wood, Sarah Cohen-Woods, Rae-Chi Huang, Colter Mitchell, Lisa Schneper, Daniel A. Notterman, Andrew J. Simpkin, Andrew D.A.C. Smith, Matthew J. Suderman, Esther Walton, Caroline L. Relton, Kerry J. Ressler, Erin C. Dunn

**Affiliations:** 1 Psychiatric and Neurodevelopmental Genetics Unit, Centre for Genomic Medicine, Massachusetts General Hospital, Boston, MA, 02114, USA; 2 Department of Psychiatry, Harvard Medical School, Boston, MA, 02115, USA; 3 Stanley Center for Psychiatric Research, The Broad Institute of Harvard and MIT, Cambridge, MA, 02142, USA; 4 Department of Epidemiology, Harvard T.H. Chan School of Public Health, Boston, MA, 02114, USA; 5 Institute for Social Research, University of Michigan, Ann Abor, MI, 48104, USA; 6 School of Population and Global Health, University of Western Australia, Crawley, WA, Australia; Menzies Research Institute, University of Tasmania, Hobart, TAS, Australia; 7 College of Education, Psychology, and Social Work, Flinders University, Adelaide, SA, Australia; 8 Flinders Institute for Mental Health and Wellbeing, Flinders University, Adelaide, SA, Australia; 9 Flinders Centre for Innovation in Cancer, College of Medicine and Public Health, Flinders University, Bedford Park, SA, Australia; 10 Nutrition Health Innovation Research Institute, Edith Cowan University, Perth, WA, Australia; 11 Department of Molecular Biology, Princeton University, Princeton, NJ, 08540, USA; 12 School of Mathematical and Statistical Sciences, University of Galway, H91 H3CY, Ireland; 13 Mathematics and Statistics Research Group, University of the West of England, Bristol, BS16 1QY, UK; 14 MRC Integrative Epidemiology Unit, Population Health Sciences, Bristol Medical School, University of Bristol, Bristol, BS8 1UD, UK; 15 Department of Psychology, University of Bath, Bath, BA2 7AY, UK; 16 McLean Hospital, Belmont, MA, 02478, USA; 17 Center on the Developing Child at Harvard University, Cambridge, MA, 02138, USA

**Keywords:** ALSPAC, longitudinal, epigenetic, DNA methylation, childhood, adversity, adolescence, trajectories, The Raine Study, FFCWS

## Abstract

**Background:**

Childhood adversity is a potent determinant of health across development. Altered DNA methylation (DNAm) signatures have been identified in children exposed to adversity and may be more common among children exposed during sensitive periods in development. However, it remains unclear if adversity has persistent epigenetic associations across childhood and adolescence. We examined the relationship between time-varying adversity and genome-wide DNAm, measured three times from birth to adolescence using prospective data from the Avon Longitudinal Study of Parents and Children.

**Methods:**

We investigated the relationship between the timing of exposure to seven adversity types (measured 5-8 times between ages 0-11) and blood DNAm at age 15 using a structured life course modeling approach. We also assessed the persistence of adversity-DNAm associations we previously identified from age 7 blood DNAm into adolescence and the influence of adversity on DNAm trajectories from ages 0-15. We attempted to replicate our age 15 associations using data from the Raine Study and Future of Families and Child Wellbeing Study (FFCWS).

**Findings:**

Adversity associated with differences in age 15 DNAm at 41 loci (R^2^≥0.035). Most loci (20/41; 49%) were associated with adversities occurring between ages 3-5. Most associations were identified for exposures to one-adult households (20/41; 49%), financial hardship (9/41; 22%), or physical/sexual abuse (4/41; 10%). Differences in age 15 DNAm were not present in age 7 DNAm; DNAm differences previously identified at age 7 resolved by age 15. We identified six distinct DNAm trajectories from these patterns of stability and persistence. We replicated the direction of associations for 90% (18/20 loci) of one-adult household loci using adolescent blood DNAm from the Raine Study and 64% of loci (18/28 loci) using saliva DNAm from the FFCWS. The direction of effects for 11 one-adult household loci were replicated in both cohorts.

**Interpretation:**

These findings highlight the time-varying impact of childhood adversity on DNAm profiles across development, providing a potential biological mechanism linking adversity to adverse health outcomes in children and adolescents.

## Introduction

Children exposed to adversity, such as abuse or maltreatment, family disruption or dysfunction, or poverty, frequently have poorer physical and mental health outcomes later in development and across the life course(1). Epigenetic processes, including DNA methylation (DNAm), are increasingly recognized as potential underlying mechanisms for these associations, as DNAm is responsive to experiences(2) and may mediate the link between environmental exposures and health outcomes(3). Indeed, hundreds of studies in humans, including population-based studies, systematic reviews, and meta-analyses have shown links between childhood adversity, DNAm, and adverse health outcomes across the life course (reviewed in (4)). However, prior studies investigating the epigenome of children exposed to adversity have not yet explored two key dimensions of the adversity-DNAm relationship: 1) the timing of adversity, and 2) the timing of DNAm measurement and its stability over time. These dimensions are critical to understand the biological risk posed by childhood adversity, identify children at risk for poor health, and improve intervention targets for health promotion and disease prevention in children and adolescents.

First, it remains unclear how the *timing* of childhood adversity might shape DNAm. Both human and animal studies suggest there may be *sensitive periods* for epigenetic programming when physiological and neurobiological systems are primed for external influences, allowing experiences to impart more enduring effects(5, 6). Notably, we have previously identified a potential sensitive period for the effects of adversity on childhood DNAm between the ages of 3-5 (7, 8). However, no prior studies have investigated sensitive periods for epigenetic patterns in adolescence.

Second, little is known about how DNAm profiles of children exposed to adversity vary across development and how DNAm variation *across time* may shape health. In a recent article, Oh and Petronis(9) argued that the dynamic nature of epigenetic mechanisms is best examined through longitudinal studies that model chrono-epigenetic patterns, meaning the dynamics of epigenetic processes across time, rather than at single timepoints. Although previous studies have shown the epigenome is dynamic across development(10–17), no study has determined how childhood adversity might influence DNAm trajectories.

To address these gaps, we examined the longitudinal relationship between early-life adversity and genome-wide DNAm across childhood and adolescence, using data collected over two decades from a subsample of youth in the Avon Longitudinal Study of Parents and Children (ALSPAC) cohort. We examined the associations between exposure to seven types of childhood adversity, assessed repeatedly between birth and age 11, and DNAm at age 15. Given the unique availability of three waves of DNAm in ALSPAC (measured from cord blood, and blood at ages 7 and 15), we also examined DNAm trajectories from birth to adolescence.

Our aims were to: 1) determine whether childhood adversity has time-dependent associations with adolescent DNAm; 2) characterize the developmental trajectories of DNAm linked to adversity; and 3) evaluate the persistence of associations between childhood adversity and DNAm at age 7 that we previously identified in ALSPAC(8) (see Figure S1 for analytic flow-chart). This study is the first to investigate the time-varying influences of childhood adversity on adolescent DNAm and DNAm trajectories from childhood to adolescence.

## Methods

### Study design and participants

ALSPAC is a large population-based birth cohort from Avon, UK of 14,451 children followed from before birth through early adulthood(18, 19). Blood-based DNAm profiles were generated for a subsample of ALSPAC mother-child pairs as part of the Accessible Resource for Integrated Epigenomic Studies (ARIES), which includes cord blood at birth (n=905), whole blood at age 7 (n=970), and peripheral blood leukocytes at age 15 (n=966)(20) (Appendix p.3).

**Figure 1:**
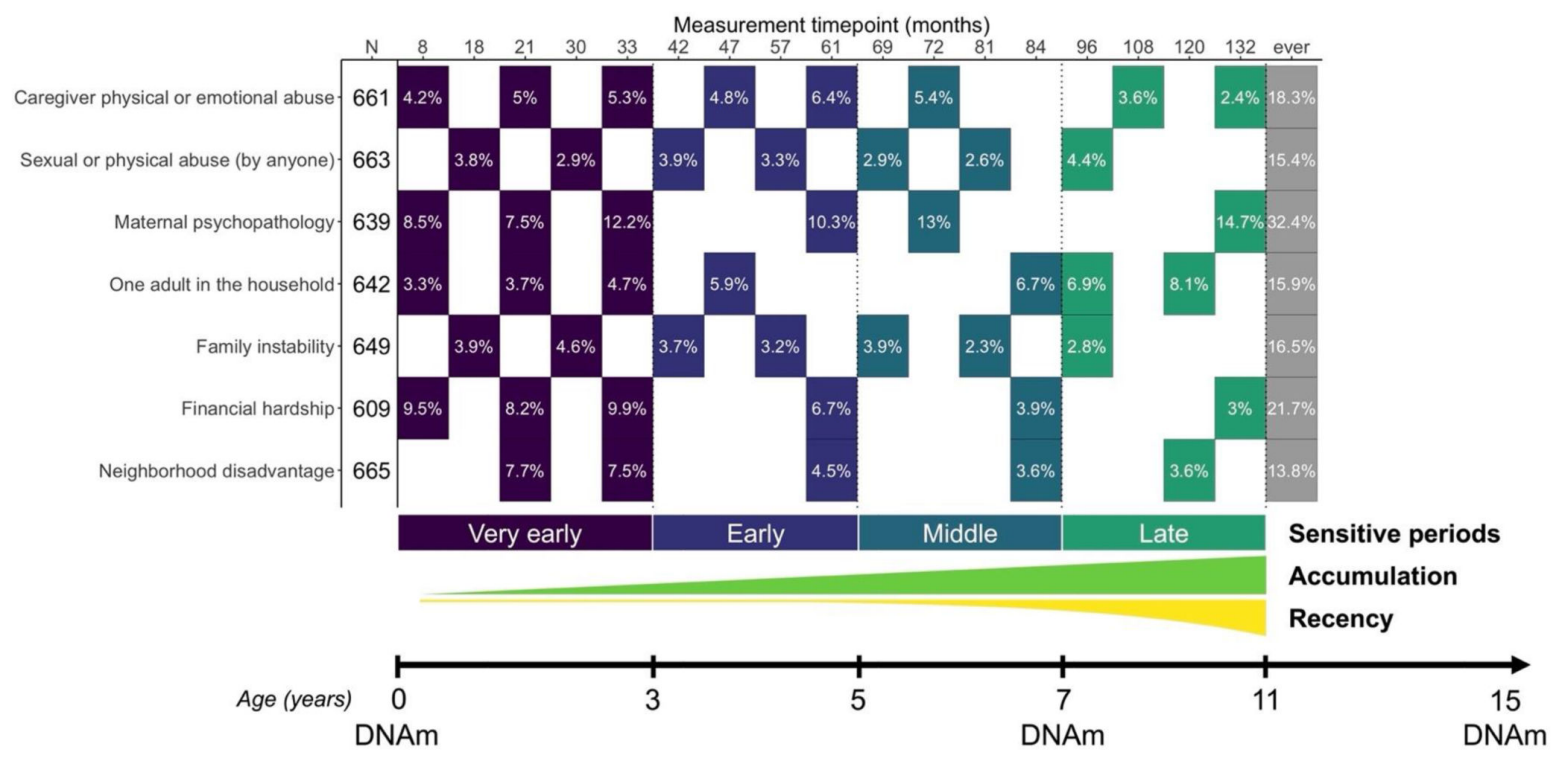
Summary of exposures and outcomes examined in the present study. Seven types of childhood adversity were assessed 5-8 times between the ages of 0 and 11. The effective sample size (N) was based on the availability of complete data for all covariates, all available timepoints of childhood adversity, and DNAm at age 15 (N=609-665). Each filled cell represents the time point when the adversity was measured, along with the prevalence of children exposed to adversity. Colors represent the four sensitive periods used to define time-dependent exposure to adversity: *very early childhood* (age 0-3), *early childhood* (age 3-5), *middle childhood* (age 5-7), and *late childhood* (age 7-11). The additional life course models tested were accumulation and recency, which reflect the total number of exposures across development and exposure to adversity weighted by time, respectively. Genome-wide DNA methylation (DNAm) data were collected at ages 0, 7, and 15.

We examined seven types of childhood adversity previously associated with DNAm: 1) caregiver physical or emotional abuse; 2) sexual or physical abuse (by anyone); 3) maternal psychopathology; 4) one-adult households; 5) family instability; 6) financial hardship; and 7) neighborhood disadvantage. These adversities were reported by mothers via mailed questionnaires, collected 5-8 times between birth and age 11 (Figure 1; Table S1).

DNAm was measured from blood at 485,577 CpG sites using the Infinium HumanMethylation450 BeadChip microarray (Illumina, San Diego, CA). Laboratory procedures, preprocessing, and quality control steps were described previously(20–21). We removed non-variable CpGs (<5% DNAm difference between children in the 10^th^ and 90^th^ percentile), resulting in 302,581 CpGs for analyses (Appendix p.3). DNAm was analyzed as beta values, which represent the percent of methylation at each site.

Ethical approval for the study was obtained from the ALSPAC Ethics and Law Committee and the Local Research Ethics Committees. Consent for biological samples has been collected in accordance with the Human Tissue Act (2004). Informed consent was obtained from participants following the recommendations of the ALSPAC Ethics and Law Committee. Secondary analyses of these data were approved with oversight by the Mass General Brigham Institutional Review Boards (Protocol 2017P001110).

### Statistical analysis

We examined time-dependent associations for each adversity among children with DNAm data and no missing data among covariates or the adversity timepoints shown in Figure 1 (N=609-665). To adjust for known potential confounders(7), we controlled for age of blood collection, sex, race/ethnicity, maternal age at birth, maternal education at birth, birthweight, number of previous pregnancies, maternal smoking during pregnancy, and cell type proportions (Appendix p.3 and Figure S2).

Our primary analyses focused on identifying time-dependent associations between each type of childhood adversity and DNAm measured in adolescence (age 15). We used the structured life course modeling approach (SLCMA), a two-stage method that simultaneously compares *a priori* life course hypotheses explaining exposure-outcome relationships(22–24). SLCMA first uses variable selection to identify the life course hypothesis explaining the greatest proportion of outcome variation. Effect estimates, confidence intervals, and p-values are then calculated for the selected life course hypothesis using post-selective inference. SLCMA detects time-varying associations with more statistical power and less bias than traditional epigenome-wide association studies of ever/never-exposed or cross-sectional paradigms (7, 8, 25).

We generated variables corresponding to six separate life course hypotheses, including four sensitive periods hypotheses encoding exposure to each childhood adversity during: 1) *very early childhood* (ages 0-2), 2) *early childhood* (ages 3-5), 3) *middle childhood* (ages 6-7), 4) *late childhood* (ages 8-11); and two additive hypotheses: 5) *accumulation of exposures* (total exposures of the specific adversity across childhood; Table S2), and 6) *recency of exposures* (total exposures of the specific adversity weighted by age) to determine whether more recent exposures had a stronger impact than distal exposures. We tested associations using selective inference and accounted for multiple-testing using the false-discovery rate (FDR). SLCMA, Quantile-quantile plots (Figure S3), genomic inflation estimates, and functional analyses of top loci are in Appendix p.4.

As sensitivity analyses, we completed internal validation analyses of the SLCMA results using ordinary nonparametric bootstrapping, and investigated the impact of potential confounders or alternate mediators of the association between childhood adversity and DNAm at age 15, including exposures to other types of childhood adversity in the same or different sensitive periods (Appendix p.5-7, 10-12).

We sought to replicate primary associations between childhood adversity and DNAm levels in adolescence using data from The Raine Study(26, 27) and the Future of Families and Child Wellbeing Study (FFCWS)(28). In the Raine Study, we analyzed the loci linked to one-adult households using blood DNAm measured at age 17 (N=382-529). In the FFCWS, we analyzed the loci linked to caregiver abuse, financial hardship, maternal psychopathology, and one-adult households using saliva DNAm measured at age 15 (N=662-1,859). The timing of adversity exposures was matched with the one identified in ALSPAC (see Appendix p.7-10).

Finally, the three waves of longitudinal DNAm data available in ALSPAC also allowed us to investigate three subsequent analyses of DNAm trajectories across development (Appendix p.12-13). First, we assessed whether DNAm differences identified at age 15 emerged earlier in development, using linear regression to test whether exposure to the same type and timing of childhood adversity was associated with DNAm at the same top loci at birth or age 7. Second, we investigated DNAm patterns in our top loci beyond the age 15 time point, studying longitudinal change and stability of DNAm across age 0, 7, and 15 among children from three distinct exposure groups: 1) children who had adversity exposure *during* the sensitive period identified from the SLCMA (labeled as exposed-SP); 2) children who had adversity exposure *outside* the sensitive period identified from the SLCMA (exposed-other); and 3) children who were never exposed to adversity.

Third, we previously identified associations between time-varying exposures to childhood adversity and DNAm levels at age 7 for 46 loci across the epigenome(8). To determine whether these DNAm alterations persisted to adolescence, we performed linear regressions between the same type and timing of childhood adversity and DNAm levels measured at age 15 for these 46 loci.

### Role of the funding sources

The funding sources played no role in the writing of the manuscript or decision to submit for publication. The authors were not paid to write this article by a pharmaceutical company or other agency.

## Results

Demographic characteristics did not differ between the ARIES sample and children exposed to any adversity between ages 0-11 (Table S3). The prevalence of exposure to a given adversity between ages 0-11 ranged from 15.1% (sexual/physical abuse, 100 of 663 children) to 34.8% (maternal psychopathology, 222 of 639 children) (Figure S4; Table S4). The tetrachoric correlation of exposure within adversity across development ranged from 0.36 (family instability) to 0.786 (one-adult households). Different types of adversity were weakly correlated (r_avg_=-0.04-0.16).

Across all types of adversity, 41 loci showed significant associations between exposure to adversity and DNAm levels at age 15 (≥3.5% of DNAm variance explained by adversity; largest p-value=5.94x10^-6^; Table 1; Table S5). Of these, 22 loci were significant after multiple-test correction (FDR<0.05). As prior studies show that p-values are poor metrics of statistical inference on their own(29, 30), particularly in the context of time-varying associations(8), we focused downstream analyses on CpGs meeting the R^2^ threshold.

**Figure 2:**
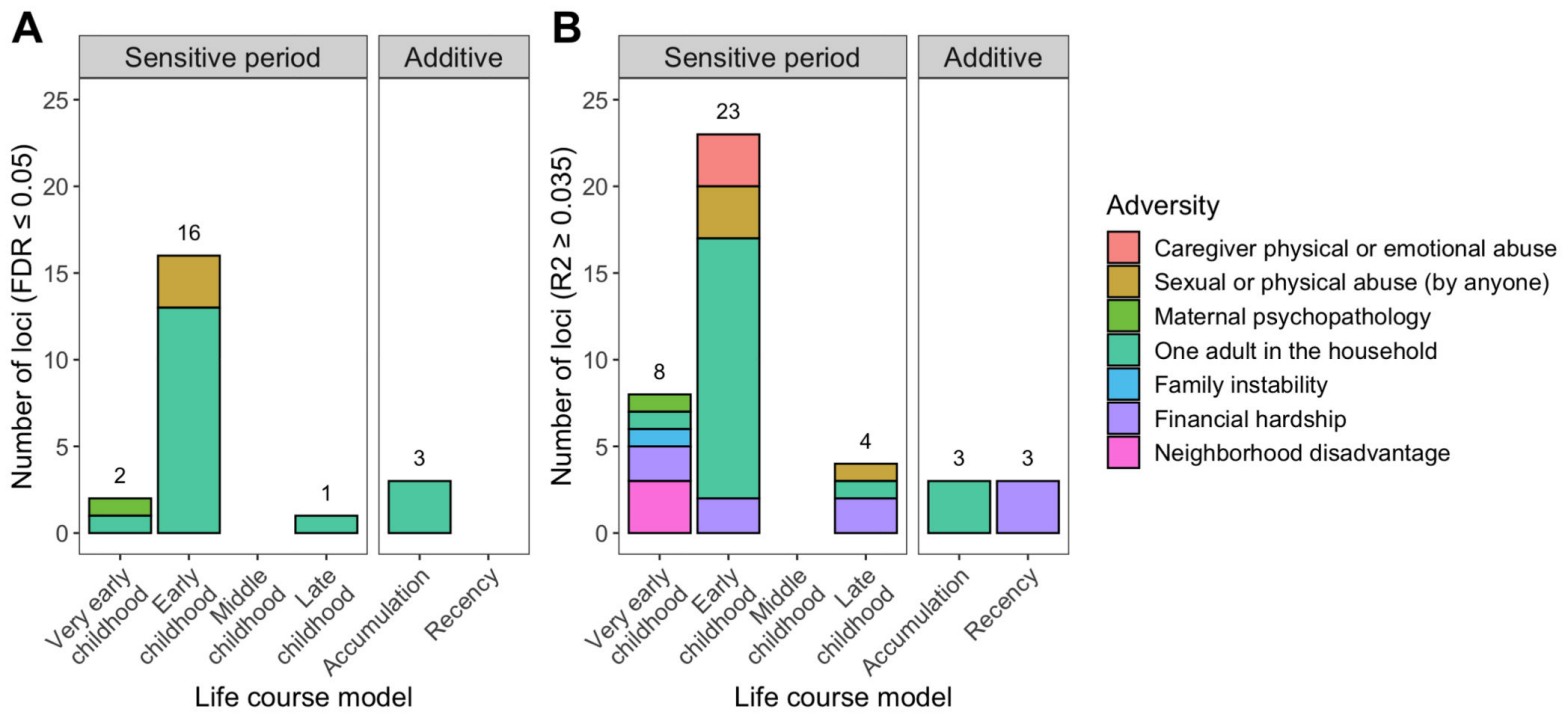
Life course theoretical models selected by the SLCMA for top loci at age 15. The life course theoretical models were split by sensitive periods (i.e., exposure to adversity during specific childhood periods) or additive models (i.e., accumulation or recency of exposures). Colors represent the different types of adversity. The distribution of theoretical models for top loci was significantly different than random chance, with exposure to adversity during sensitive periods more frequently predicting DNA methylation levels as compared to the additive models. **A)** 22 loci were identified at a false-discovery rate (FDR) <0.05. Most loci were associated with exposure to one-adult households during early childhood. **B)** 41 loci were identified at an R^2^≥0.035 cutoff and p<1x10^-5^ threshold, which again mainly showed associations with adversity occurring during early childhood.

Sensitive periods were the most often selected life course hypothesis by the SLCMA, with 35 loci showing associations with childhood adversity that occurred during *very early childhood* (20%; 18/41), *early childhood* (56%; 23/41), or *late childhood* (10%; 4/41) (Figure 2). Only 3 loci (7%) showed associations with the accumulation or recency of adversity. Most of these associations were for exposure to one-adult households (20 loci), followed by financial hardship (9 loci), sexual or physical abuse by anyone (4 loci), caregiver physical or emotional abuse (3 loci), neighborhood disadvantage (3 loci), family instability (1 locus), and maternal psychopathology (1 locus).

Childhood adversity was mainly associated with a decrease in DNAm (35/41 loci). On average, childhood adversity exposure was linked to a 3.5% absolute difference in DNAm (range 0.9-10.4%). For loci associated with accumulated time living in one-adult households, each additional exposure timepoint associated with a 1% difference in DNAm (range 0.3-1.4%). For loci associated with the recency of financial hardship, one additional exposure was linked to a - 1.3% to 2.3% change in DNAm per year of age at exposure.

Top loci showed higher representation in low CpG density regions, such as enhancers (p=0.008) and Open Seas (p=0.018) (Figure S5). Most loci (28/41) had weak, positive brain-blood correlations in individuals without exposure to adversity (28/41 positive; ravg=0.10; 10 with p<0.05; Table S6; Figure S6)(31), suggesting adversity-associated differences in blood DNAm could be reflected in the central nervous system. No biological processes were significantly enriched in top loci using the DAVID or *missMethyl* gene ontology tools(32, 33)(Figures S7- S8). Seven genes linked to sexual/physical abuse (*TAF1*), family instability (*PKD2*), financial hardship *(FBXL16, XKR6)*, or one-adult households *(DSP, CUX2, STK38L)* showed evidence of strong functional constraint through analyses of probability of intolerance to loss-of-function mutations(34)(Table S5; Figure S9). Finally, several loci were previously associated with gestational age (7 loci), sex (6 loci), smoking (1 locus), inflammatory bowel disease (1 locus), and rheumatoid arthritis (4 loci). Together, these findings suggest different types of childhood adversity may act through diverse biological processes (Appendix p.4-5).

Internal validation of top associations yielded nearly identical results to the initial analyses (largest difference in effect estimates=2.03%) (Figure S10; Table S7). Our results remained stable when correcting for exposure to other adversities during the sensitive period or across childhood, suggesting they were not influenced by co-occurring adversity (Appendix p.6-7; Figure S11-13). Together, these results point to the robustness and specificity of associations between time-varying childhood adversity and DNAm at age 15.

We attempted to replicate these associations in two independent datasets, the Raine Study and FFCWS (Figure S14). Using data from the Raine Study (blood DNAm), we tested associations for the 20 CpGs associated with one-adult households (Table S8). Of these, 18 CpGs (90%) showed the same direction of effects in the Raine Study, which was more likely than random chance (p=2x10^-4^; Figure S15). Three CpGs were nominally significant (p<0.05) in the Raine Study; none of the effect estimate confidence intervals contained zero and all had the same direction as ALSPAC. Effect estimates in the Raine Study were smaller compared to ALSPAC. These differences were mitigated when correcting for winner’s curse effects (Figure S15).

Using data from FFCWS (saliva DNAm), we attempted to replicate associations for 28 loci associated with four childhood adversities. Of these, 64% of CpGs (18/28) showed the same direction of effects in the FFCWS (p=0.092), with 73% of one-adult household loci (11/15) showing concordant directions (p=0.059; Figure S16; Table S9). Importantly, all 11 of these one-adult household loci showed the same direction of effects in the Raine Study. While the magnitudes of effects were smaller in FFCWS, one CpG associated with the accumulation of one-adult household exposures (cg00807464; *CUX2)* showed nearly identical effect estimates between cohorts. These results point to the partial replication of associations from ALSPAC in independent cohorts, particularly for exposures to one-adult households.

**Figure 3:**
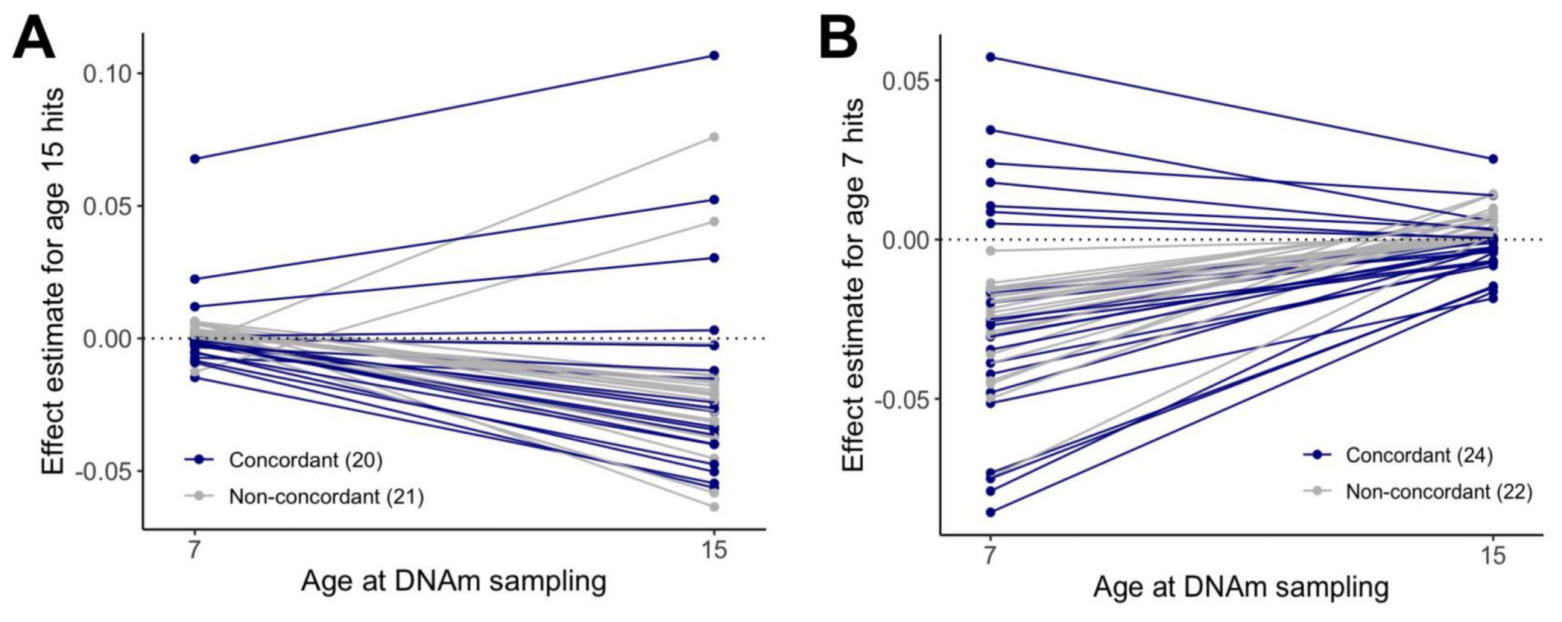
Persistence and stability of associations between childhood adversity and DNA methylation across development. **A)** The estimates of associations between childhood adversity and DNAm at age 7 or age 15 generally showed variable directions-of-effect for the significant loci identified from the SLCMA at age 15 (20 concordant and 21 non-concordant directionality). Estimates for age 7 DNAm data were also smaller than those at age 15, suggesting that these loci showed latent responses to adversity. **B)** The estimates of associations between childhood adversity and DNAm at age 7 or age 15 generally showed variable directi ons-of-effect for the significant loci identified in a previous study of age 7 DNAm (24 concordant and 22 non-concordant directionality). Estimates for age 15 DNAm data were also smaller than those at age 7, suggesting that these loci showed early responses to adversity that resolved by adolescence.

For the 41 loci identified in age 15 DNAm, none showed associations between adversity and DNAm at birth (Table S10) or age 7 (Table S11). Notably, the age 7 estimates were *smaller* than the age 15 associations, with consistent directions-of-effect in about half of loci (20/41) (Figure 3A). Agnostic of adversity exposure, correlations in DNAm levels across ages were low at the individual-level (r_avg_=0.11; Figure S17). The emergence of these associations was not explained by early-life confounders (<10% change in effect estimates for parental socio-economic position, maternal BMI, or gestational age) or biological mediators during adolescence (<5% of the association mediated through age at pubertal onset, adolescent BMI, CRP levels, or smoking), suggesting some adolescent differences may emerge later in development and become stronger with time (Appendix p.10-12); Figures S2, S18-24).

**Figure 4:**
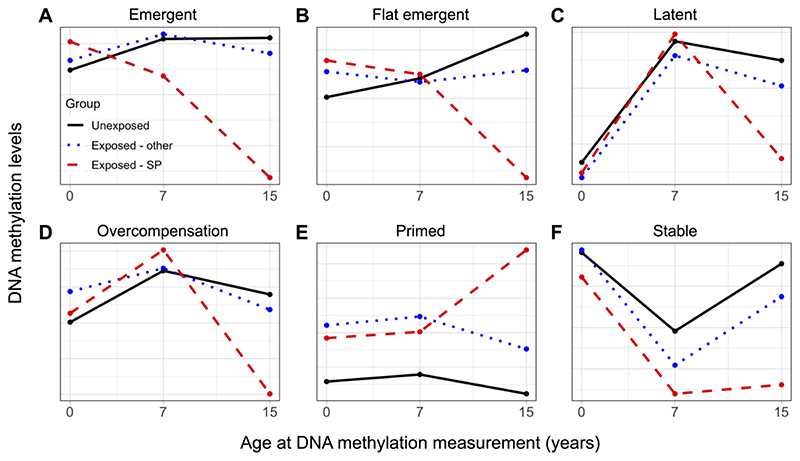
DNA methylation trajectories across development. Distinguishing features included DNAm differences emerging earlier versus later in development, differences between children exposed during a sensitive period (exposed-SP) or at other developmental stages (exposed-other), and differences linked to age at DNAm measurement. **A)** Emergent trajectory (5 loci): differences in exposed- SP appeared in childhood but did not fully emerge until age 15. **B)** Flat emergent trajectory (2 loci): differences in exposed-SP were modest throughout childhood and fully emerged by age 15. **C)** Latent trajectory (17 loci) differences for exposed-SP emerged at age 15, with no differences observed from exposure at other times. Some CpGs in this cluster showed graded differences between childhood exposed in sensitive periods versus other times. **D)** Overcompensation trajectory (9 loci): cross-over of DNAm differences in exposed-SP were present from age 7 to age 15, along with differences in DNAm level between ages. **E)** Primed trajectory (1 loci): differences in the exposed groups were apparent from birth but were magnified in exposed-SP at age 15. **F)** Stable trajectory (7 loci): differences in exposed-SP were present at age 7 and remained stable until age 15.

Moving beyond adolescent DNAm, 34 of the 41 loci had significant adversity exposure group-by-age interactions (FDR<0.05), suggestive of more complex patterns of change and stability across development. From these loci, we identified five additional types of longitudinal DNAm trajectories (Figure 4), which showed distinct DNAm patterns across ages and adversity exposure groups (Figures S25-28; Table S12), but not between the FDR and R^2^ subsets of CpGs (Figure S29).

Finally, of the 46 CpG sites previously showing time-varying associations between adversity and DNAm at age 7 (8), only one showed an association at age 15 (p<0.05; Table S13), which did not pass multiple-test correction. Again, approximately half of loci showed consistent direction-of-effect between age 7 and 15 (24/46) (Figure 3B). These findings suggest some childhood epigenetic responses to adversity may not persist into adolescence.

## Discussion

This study’s main finding is that associations between childhood adversity and DNAm vary across the life course, manifesting at different developmental stages through distinct patterns of persistence and latency. To our knowledge, this is the first study to incorporate time-dependent measures of childhood adversity when assessing longitudinal epigenetic patterns.

Our findings point to early childhood – the period between ages 3 to 5 – as a possible sensitive period for the biological embedding of childhood adversity that manifests in adolescence. These findings are consistent with prior human and animal studies showing that exposures earlier in life may have greater influence on epigenetic patterns measured in childhood(7, 8) or adolescence(35). As early childhood is a time of rapid cognitive, social, emotional, and regulatory development(36), epigenetic processes may be more malleable(12), resulting in increased sensitivity to life experiences that shape DNAm levels and trajectories across development. These findings suggest early childhood may be a period for focused interventions to limit or prevent the long-term sequelae of childhood adversity.

Of the seven types of adversity examined, exposure to single parent households had the greatest number of associations to DNAm in adolescence. By contrast, previous research on DNAm from the same children at age 7 identified no associations with one-adult households(8), suggesting these associations are adolescent-specific. Prior studies have shown the effects of single parent households begin to emerge around puberty, manifesting through shifts in puberty timing (37), poorer self-esteem(38), and higher depressive symptoms(39) and externalizing behaviors(39). Of note, we did not detect any mediation of the associations of one-adult households and DNAm through pubertal onset age, nor were any loci previously linked to pubertal onset or sex hormone levels, or confounded by socioeconomic factors (Figure S19). We also replicated the direction of associations for 11 loci associated with one-adult households in two independents cohort. These results are particularly salient given the differences in the sociodemographic contexts and in the DNAm tissue assessed between studies. Beyond broad tissue differences, saliva is more heterogenous across individuals than blood (40), which further increased the stringency of the replicated effects and highlights the potential relevance of these top loci. Overall, these findings suggest a latency to the effects of one-adult households on biological processes and health outcomes, which may not become apparent until the rapid developmental changes occurring during puberty.

Curiously, we observed fewer associations for other adversities, such as maternal psychopathology and experiences of sexual, physical, or emotional abuse. These adversities may have subtler influences on the adolescent epigenome, requiring larger sample sizes or meta-analyses to uncover. None of our top loci overlapped between different types of childhood adversity, nor were they present among top loci from a twin study of adolescents exposed to severe victimization (N=118)(11). As discussed in ongoing debates surrounding the “lumping or splitting” of childhood adversities in clinical research(41), different dimensions of adversity could result in distinct epigenetic signatures, a hypothesis supported by the finding that adjusting for other types of adversity only modestly influenced associations. Of note, we found that exposures to deprivation-type adversities during early childhood may have more influence on adolescent DNAm than threat-type adversities (42)(Figure S30).

Arguably the most novel finding from our study concerned the patterns of stability and change in the relationship between adversity and DNAm. Most DNAm trajectories showed primarily *latent* associations with adversity, meaning they did not emerge until age 15 in youth exposed to adversity. These findings align with previous longitudinal studies of genome-wide DNAm from ALSPAC and Project Viva, which showed that early-life stressors, such as prenatal maternal smoking(13) and socio-economic disadvantage during childhood(10, 14), can have both immediate and latent associations with DNAm during childhood and adolescence. Subtle desynchronization of DNAm levels may appear earlier in development, while evading immediate detection until later in life. These “sleeper” patterns may explain why complex diseases unfold over years of development, rather than immediately after exposures or risk factors(9). We also note that most of our top loci showed little individual-level stability over time, suggesting these latent effects may be located within regions of the epigenome that change across development.

Future research is needed to determine whether latent associations between childhood adversity and the epigenome persist into adulthood and whether they are more likely to influence physical and mental health than alterations arising earlier in development.

Similarly, the DNAm differences we previously observed at age 7 did not persist into adolescence(8). Studies on early-life stressors(10, 14), birthweight and gestational age(16), and maternal weight before and during pregnancy(15) parallel these findings, showing that DNAm differences linked to early-life environments rarely persist across time. Whether these patterns resolve naturally or due to active intervention is unknown and should be investigated to determine whether interventions can be beneficial in reversing epigenetic effects of early-life stressors. Nevertheless, even short-term alterations that eventually fade over time could alter the developmental trajectories of downstream cellular pathways to influence future health.

Several differentially methylated genes we identified were implicated in processes that could influence downstream disease. For instance, *CUX2* is transcription factor involved in dendrite and synapse formation(43), alterations to which could influence neurodevelopment and vulnerability to mental disorders. Several top genes, including *DUSP10, DSP*, and *VEGFA*, are also linked to cardiac function, and may partially reflect mechanisms linking childhood adversity to heart disease(44). We note, however, that findings from epigenome and genome-wide association studies have different interpretations and have not yet converged on common mechanisms underlying human health and disease. As DNAm alterations may not reflect concomitant changes in gene function or expression, experimental studies are needed to identify the true functional and health consequences of these epigenetic differences and determine whether short- and/or long-term DNAm changes could link childhood adversity to adverse health outcomes across the lifespan.

If replicated, our results may reveal how the biological embedding of early-life exposures through DNAm contribute to disease risk across development, which could have important clinical implications for early risk prediction, disease prognosis, and therapeutic guides for individuals and populations exposed to adversity. Several recent studies have shown that DNAm can predict risk and progression of diseases such as cancer(45) and depression(46). It may be that certain adversity-associated DNAm trajectories predict concomitant trajectories of disease risk. If true, repeated measures of DNAm could serve as a biological indicator or early warning-sign of initiated disease processes, helping identify people at greater risk for future disease. Moreover, these adversity-associated DNAm trajectories may also act as biological measures of treatment response, for example to salutary interventions or protective factors designed to buffer against the effects of adversity. Recent research shows that DNAm differences among adults with post-traumatic stress disorder (PTSD) (compared to those without PTSD) resolved following psychotherapy treatment; such DNAm changes corresponded to a reduction in PTSD symptom severity(47). Thus, repeated measures of DNAm could be used as a marker of therapeutic efficacy, tracking possible disease progress and/or resolution.

Our study had limitations. First, DNAm data were generated from slightly different tissue types at each wave. Although we corrected for cell type composition using established methods, differences in the stability of DNAm differences between waves may have been partially driven by tissue-based differences and variability. Second, we could not replicate all findings, partially due to the lack of available data from the Raine Study and FFCWS. Further, differences in associations between cohorts could reflect differences in the socio-economic environment or the specific timing and tissue of DNAm measurements, among other factors. Future studies should confirm these longitudinal epigenetic responses to childhood adversity and triangulate the socio-biological factors that modulate adversity-induced epigenetic differences and health outcomes. Third, we cannot rule out the possibility that unmeasured confounding or technical factors influenced our findings. However, our results were robust in internal validation analyses and when controlling for 11 potential confounders and investigating four potential mediators. Similarly, we could not assess the impact of time-varying confounding, which could have influenced our results(48). Fourth, our analytic subsample was mainly composed of children from European descent. This lack of diversity limited the generalizability of our findings, emphasizing the importance of replicating this work in more diverse cohorts. Finally, the differences in DNAm observed in youth exposed to adversity may not reflect concomitant phenotypic alterations, as epigenetic alterations in peripheral tissues may only partially reflect the causal mechanisms that drive health and disease. Thus, studies that combine both model systems and human populations are necessary to fully delineate the relationships among adversity, DNAm, and health.

In sum, this study highlights developmental variability in the relationship between adversity and DNAm trajectories and its potential role in adversity-related health outcomes across childhood and adolescence. Future studies should continue to investigate longitudinal measures of DNAm to identify the potential role of latent and persistent epigenetic alterations in driving the short- and long-term health outcomes that result from childhood adversity. Ultimately, this research will help guide intervention strategies and identify individual at higher risk for physical and mental disorders arising from exposure to childhood adversity.

## Supplementary Material

Supplement

## Figures and Tables

**Table 1:** Top associations between time-dependent exposure to adversity and DNA methylation at age 15.

Adversity	Timing	Age (years)	CpG	DNAm unexp^1^	DNAm SP^2^	Δ DNAm^3^	Effect estimate^4^	SE*	95% CI*	R2^5^	P-value	FDR- adjusted p-value	Nearest gene	Trajectory class
Caregiver physical or emotional abuse	Early childhood	5	cg14855874	0.091	0.121	0.030	0.030	0.005	0.019; 0.041	0.041	3.32E-07	1.01E-01	BANK1	Emergent
			cg15454534	0.885	0.868	-0.017	-0.017	0.003	-0.023; -0.01	0.039	6.76E-07	1.02E-01	OR2T1	Latent
			cg06215562	0.847	0.826	-0.021	-0.021	0.004	-0.029;-0.013	0.035	2.37E-06	1.81E-01		Latent
Sexual or physical abuse by anyone)	Early childhood	3.5	cg26970800	0.902	0.847	-0.055	-0.055	0.010	-0.074; -0.036	0.044	8.51E-08	2.08E-02	CBLIF	Emergent
			cg15723468	0.822	0.779	-0.043	-0.045	0.009	-0.062; -0.028	0.041	1.89E-07	2.08E-02	GALNT2	Latent
			cg17928317	0.681	0.785	0.104	0.076	0.015	0.045; 0.106	0.041	2.06E-07	2.08E-02	MAGEC3	Primed
	Late childhood	8	cg27558057	0.257	0.289	0.032	0.107	0.024	0.059; 0.155	0.036	1.53E-06	1.16E-01	TAF1	Stable
Family instability	Very early childhood	2.5	cg02735620	0.877	0.857	-0.021	-0.019	0.004	-0.027; -0.012	0.036	2.07E-06	4.63E-01	PKD2	Emergent
Financial hardship	Very early childhood	0.66	cg14455319	0.289	0.339	0.050	0.052	0.011	0.032; 0.074	0.036	3.87E-06	2.00E-01	ANKK1	Time-stable
			cg13204236	0.861	0.824	-0.037	-0.037	0.007	-0.051; -0.023	0.036	5.94E-06	2.00E-01	STPG4	Latent
	Early childhood	5	cg15037420	0.780	0.746	-0.035	-0.034	0.007	-0.049; -0.021	0.036	3.04E-06	2.00E-01	BSPH1	Latent
			cg06410970	0.860	0.825	-0.035	-0.033	0.006	-0.046; -0.022	0.036	5.56E-06	2.00E-01	ANXA11	Overcompensation
	Late childhood	11	cg02011706	0.861	0.799	-0.062	-0.064	0.013	-0.089; -0.039	0.036	5.35E-06	2.00E-01	LMF1	Emergent
			cg04659536	0.901	0.873	-0.029	-0.028	0.006	-0.039; -0.017	0.035	5.52E-06	2.00E-01	SDK1	Latent
	Recency		cg17670999	0.817	0.807	-0.010	-0.002	0.000	-0.003; -0.001	0.041	8.76E-07	2.00E-01	ARHGAP39	Stable
			cg25459301	0.769	0.756	-0.013	-0.003	0.001	-0.004; -0.002	0.036	4.24E-06	2.00E-01	XKR6	Overcompensation
			cg06812747	0.837	0.825	-0.012	-0.003	0.001	-0.004; -0.002	0.035	4.98E-06	2.00E-01	FBXL16	Stable
Maternal psychopathology	Very early childhood	2.75	cg16813552	0.898	0.883	-0.015	-0.015	0.003	-0.021; -0.01	0.045	7.11E-08	2.15E-02	OGA	Stable
Neighborhood disadvantage	Very early childhood	2.75	cg04288299	0.914	0.905	-0.009	-0.021	0.004	-0.029; -0.013	0.039	4.52E-07	7.00E-02	NELFA	Overcompensation
			cg25019631	0.201	0.223	0.023	0.044	0.009	0.028; 0.061	0.038	6.16E-07	7.00E-02	CASP9	Overcompensation
			cg04224851	0.907	0.894	-0.013	-0.014	0.003	-0.02; -0.009	0.038	6.94E-07	7.00E-02	ZFP36L2	Overcompensation
One adult in the household	Very early childhood	1.75	cg05491478	0.908	0.880	-0.028	-0.027	0.006	-0.039; -0.016	0.038	7.33E-07	2.81E-02	LRRFIP1	Overcompensation
	Early childhood	3.9	cg16907527	0.853	0.824	-0.030	-0.032	0.005	-0.041; -0.022	0.060	4.17E-10	1.26E-04	VEGFA	Flat emergent
			cg08818094	0.847	0.798	-0.048	-0.050	0.008	-0.067; -0.034	0.051	8.79E-09	1.33E-03	TBC1D19	Latent
			cg01060989	0.824	0.794	-0.031	-0.031	0.005	-0.042; -0.021	0.047	6.73E-08	6.78E-03	DUSP10	Latent
			cg15814750	0.723	0.684	-0.039	-0.040	0.009	-0.058; -0.025	0.039	6.57E-07	2.81E-02	WDR72	Latent
			cg15783822	0.868	0.848	-0.021	-0.021	0.004	-0.031; -0.014	0.039	8.08E-07	2.81E-02	PRR4	Latent
			cg15864691	0.907	0.889	-0.018	-0.018	0.004	-0.025; -0.011	0.038	8.36E-07	2.81E-02	HOXA10	Overcompensation
			cg02584161	0.661	0.603	-0.057	-0.058	0.011	-0.081; -0.038	0.038	1.28E-06	3.42E-02		Latent
			cg02810291	0.840	0.818	-0.022	-0.023	0.005	-0.033; -0.014	0.037	1.35E-06	3.42E-02	AKAP13	Overcompensation
			cg04036644	0.882	0.855	-0.027	-0.026	0.005	-0.037; -0.016	0.037	1.36E-06	3.42E-02	LOC286083	Latent
			cg11811897	0.758	0.711	-0.047	-0.047	0.010	-0.067; -0.03	0.037	1.68E-06	3.64E-02	PKD1L1	Latent
			cg15817130	0.794	0.759	-0.036	-0.038	0.007	-0.051; -0.025	0.037	1.83E-06	3.69E-02	MYO10	Latent
			cg06711254	0.686	0.631	-0.055	-0.056	0.012	-0.08; -0.036	0.036	2.15E-06	3.98E-02	FSIP2	Flat emergent
			cg19096460	0.845	0.821	-0.024	-0.024	0.005	-0.035; -0.015	0.035	2.89E-06	4.85E-02	HERC3	Latent
			cg18980650	0.800	0.760	-0.040	-0.036	0.007	-0.05; -0.024	0.035	3.31E-06	5.08E-02	NOX1	Emergent
			cg27504269	0.771	0.733	-0.038	-0.040	0.008	-0.056; -0.026	0.036	3.52E-06	5.08E-02	SLCO1A2	Latent
	Late childhood	10	cg12096528	0.890	0.874	-0.016	-0.016	0.003	-0.023; -0.01	0.036	2.24E-06	3.98E-02	SLC25A41	Overcompensation
	Accumulation		cg00807464	0.052	0.057	0.006	0.003	0.001	0.002; 0.004	0.040	7.56E-07	2.81E-02	CUX2	Stable
			cg10420609	0.559	0.522	-0.037	-0.014	0.003	-0.02; -0.009	0.039	7.71E-07	2.81E-02	DSP	Latent
			cg14579651	0.634	0.605	-0.028	-0.012	0.002	-0.018; -0.008	0.037	1.68E-06	3.64E-02	STK38L	Stable

1 DNAm unexp. = mean DNA methylation levels in children with no exposure to adversity from ages 0 to 11.

2 DNAm exp. SP = mean DNA methylation levels in children with exposure to adversity that occurred during the selected sensitive period (SP). Accumulation hypotheses show the mean DNA methylation levels in children with at least one exposure to adversity.

3 △DNAm= difference in mean DNA methylation levels between children exposed to adversity during the selected sensitive period and individuals unexposed to adversity (i.e., DNAm exp. SP – DNAm unexp.)

4 Effect estimates were calculated using linear regression of exposure to adversity from the theoretical model and DNA methylation, correcting for the covariates described in the methods. Standard error and confidence intervals are shown for these estimates.

5 R^2^ is the proportion of variation in DNAm at this CpG that is explained by differences in this adversity at this timing, after removing the associations with covariates.

* CI = Confidence Interval; SE = standard error; Very early childhood = 0-3 years, Early childhood = 3-5 years; Late childhood = 8-11 years.

## Data Availability

ALSPAC data are available by request from the ALSPAC Executive Committee for researchers who meet the criteria for access to confidential data (bristol.ac.uk/alspac/researchers/access/). Data from the Raine Study are available with the permission of the Raine Study. Restrictions apply to the availability of these data, which were used under license for this study. The FFCWS data analyzed in the current study are available with permission from the Future of Families and Childhood Wellbeing Study repository (fragilefamilies.princeton.edu/documentation)

## Research In Context

### Evidence before this study

We searched PubMed from inception to July 29, 2022 for articles on childhood adversity and DNA methylation measured during childhood and adolescence in human populations. Search terms included “DNA methylation OR epigenetics”, “trauma OR adversity OR abuse”, “child OR childhood”, “adolescent OR adolescence”. Our search did not identify any previous studies that investigated time-varying associations between childhood adversity on adolescent DNA methylation or trajectories of DNA methylation across development.

### Added value of this study

To our knowledge, this is the first human study to incorporate time-dependent measures of childhood adversity in the study of longitudinal epigenetic patterns. Our findings are the first to demonstrate the dynamic developmental associations between adversity on the human epigenome. These analyses extend prior work that revealed sensitive periods for the association of childhood adversity with epigenetic alterations at age 7 in ALSPAC, further highlighting that exposure to adversity between the ages of 3-5 may be more closely linked to biological processes and future health than exposure during other time periods.

### Implications of all the available evidence

Our study suggests epigenetic mechanisms may serve as a biological link between childhood adversity and long-term health. If replicated, these findings could explain why there are both immediate and latent manifestations of disease among people with histories of childhood adversity. Our findings also support the need for further studies investigating the role of DNA methylation trajectories in predicting child and adolescent health, including risk for immune dysfunction, metabolic disorder, and mental health problems.
